# Job‐Related Stressors and Quality‐Of‐Life Enjoyment and Satisfaction Across Nurses' Career Stages: A Multicenter Cross‐Sectional Study

**DOI:** 10.1002/nop2.70978

**Published:** 2026-11-02

**Authors:** Hind Ismail Ali, Safaa Moustafa Mohamed Abdeen, Hanaa Sayed Ewise

**Affiliations:** ^1^ Department of Nursing, College of Applied Medical Sciences Prince Sattam Bin Abdul Aziz University Wadi ad‐Dawasir Saudi Arabia; ^2^ Faculty of Nursing Sohag University Sohag Egypt

**Keywords:** career stages, employment, nurses, occupational stress, quality‐of‐life enjoyment and satisfaction

## Abstract

**Aim:**

To assess job‐related stressors among nurses across different career stages and examine their relationships with quality‐of‐life enjoyment and satisfaction.

**Design:**

A multicenter, descriptive‐correlational, cross‐sectional research design.

**Methods:**

This study was conducted in nine hospitals were geographically distributed across different sectors of the Sohag governorate 1 August to 30 September 2025. The study participants included 277 nurses at different career stages. Data were collected using: General Information Questionnaire, the Work Stress Assessment Questionnaire, and the Quality‐of‐Life Enjoyment and Satisfaction Questionnaire Short Form.

**Results:**

Job‐related stress was relatively high, with Reward identified as the prominent stressor, while Quality‐of‐life enjoyment and satisfaction was generally low. Significant differences in stress and quality of life were observed across age groups, nursing roles, and career stages mainly at during the establishment stage, followed by the exploration stage, whereas gender and marital status showed no significant differences. A strong negative correlation was found between job‐related stress and quality‐of‐life enjoyment and satisfaction.

**Conclusion:**

Nurses experienced different job‐related stressors across their career stages particularly for early‐ and mid‐stages. Also, their level of quality‐of‐life enjoyment and satisfaction enhanced across career stage. This is highlighting the association of job‐related stress on nurses' quality‐of‐life enjoyment and satisfaction and the need for targeted interventions.

**Implications for Nursing:**

Nurse managers should implement career‐stage‐specific stress management, mentoring, and well‐being programs to enhance nurses' enjoyment and satisfaction and job retention.

**Implications for Health Policy:**

Healthcare policies should prioritize psychologically healthy work environments, adequate staff empowerment and recognition, and career development initiatives that reduce occupational stress and promote nurses' well‐being.

**Abbreviations**
ICN: International Council of Nurses
JD–R: Job Demands–Resources
PERMA: Positive emotions, Engagement, Relationships, Meaning, and Accomplishment
Q‐LES: Quality of Life Enjoyment and Satisfaction
Q‐LES‐Q‐SF: Quality‐of‐Life Enjoyment and Satisfaction Questionnaire Short Form
STROBE: Strengthening the Reporting of Observational Studies in Epidemiology
WSAQ: Work Stress Assessment Questionnaire

## 1 Introduction

Recent systematic evidence indicates that healthcare professionals experience substantial occupational demands, including high workload and demanding work conditions, which can adversely affect physical and emotional well‐being. A systematic review of 16 studies found that excessive work demands among medical personnel were associated with burnout and mental‐health problems, including depression, anxiety, and abnormal stress (Aprilia et al. 2024). These findings underscore that occupational stress represents a broader challenge across healthcare professions, providing an important context for examining nurses, who constitute a large and continuously exposed component of the healthcare workforce. Nursing is widely recognized as a highly demanding profession, with job stressors linked to reduced quality of care, burnout, turnover, and diminished job satisfaction (Clark and Lake 2020; Roczniewska and Bakker 2021). Burnout affects 20%–40% of nurses, undermining well‐being and organizational outcomes. Stressors such as staff shortages, heavy workloads, exposure to death and dying, and strained relationships with supervisors contribute to emotional exhaustion and increased turnover intentions (Elliason 2022; Kilpatrick 2024). The International Council of Nurses (2025) highlights the economic burden of stress and burnout, estimating billions in lost productivity, while interventions to improve nurse well‐being could restore substantial value (Hassard et al. 2018; Williams 2025).

Career stage models further illustrate how stress and coping demands vary across the professional lifespan. Nurses' careers generally progress through four stages defined by professional experience years. The exploration stage (0–5 years) involves transitioning from academic preparation to clinical practice, developing foundational skills, and adapting to workplace demands, often accompanied by uncertainty and anxiety (Super 1988; Bai and Liu 2018). The establishment stage (5–10 years) is characterized by growing stability, peer acceptance, and refinement of clinical competence. The advancement stage (10%–20%) reflects mid‐career consolidation, specialization, and peak productivity, often accompanied by leadership responsibilities and recognition (Bai and Liu 2018). Finally, the maintenance stage (20+ years) represents late‐career development, where nurses focus on mentoring, preparing successors, and balancing professional duties with personal interests, finding fulfilment in guiding younger colleagues and sustaining organizational stability (Mainiero and Sullivan 2005; Scarbrough 2023).

The National Institute for Occupational Safety and Health (NIOSH) defines job stress as ‘the harmful physical and emotional responses that occur when the requirements of the job do not match the capabilities, resources, or needs of the worker’ (Sauter et al. 1999). Recently, job‐related stress is described as the negative psychosomatic and physiological response which occurs when work requests surpass the individuals' ability to handle those demands (Daniel 2019). Although moderate levels of stress are believed to enhance creativeness and effort, excessive levels of stress result in decreased creativeness and effort (International Labour Organization 2020). Decide that Job stressors could influence not only professional outcomes but physical health, emotional well‐being, social relationships, and daily functioning. Instruments such as the Quality‐of‐Life Enjoyment and Satisfaction scale (Endicott et al. 1993; Stevanovic 2011; Abbaszadeh et al. 2024) provide valuable insights into how workplace experiences shape nurses' overall well‐being. Understanding these dynamics is essential for designing interventions that support nurses at different career stages and enhance both retention and thriving at work.

The Job Demands–Resources (JD–R) model proposes that demands such as workload, emotional strain, and organizational pressures increase stress, whereas resources such as leadership support, teamwork, autonomy, and professional development promote engagement and resilience (Bakker and Demerouti 2024). The PERMA model conceptualizes well‐being through Positive emotions, Engagement, Relationships, Meaning, and Accomplishment (Seligman 2018). Consequently, variations in job demands and available resources across career stages may affect nurses' quality‐of‐life enjoyment and satisfaction, including their perceived satisfaction with work, relationships, physical and emotional well‐being, and daily activities. Career stage may modify this balance as nurses' responsibilities, competence, autonomy, and professional expectations evolve. Early‐career nurses may face greater adjustment demands and require more support, whereas mid‐ and late‐career nurses may experience increased autonomy, specialization, leadership responsibilities, and mentoring roles (Super 1988; Bai and Liu 2018; Scarbrough 2023). Thus, integrating career stage into the JD–R and PERMA frameworks provides a basis for understanding how job‐related stressors may differentially influence nurses' quality‐of‐life enjoyment and satisfaction across their career stages.

Job‐related stress is a major challenge for healthcare systems worldwide and may adversely affect nurses' quality‐of‐life enjoyment and satisfaction. In a 2022 survey of 11,964 nurses, more than 70% reported experiencing stress recently (American Nurses Foundation 2022). Similarly, studies conducted in Egypt have reported high levels of job‐related stress and burnout among nurses and their associations with job dissatisfaction, psychological strain, and turnover intention (Said and El‐Shafei 2021; Ibrahim et al. 2025). Although previous research has examined job‐related stress across different nursing positions (Chang et al. 2021), no evidence has explored how specific job‐related stressors vary across nurses' career stages. Moreover, the relationship between job‐related stress and quality‐of‐life enjoyment and satisfaction has received limited attention in nursing research, particularly in Upper Egypt. This gap is important because nurses at different career stages may encounter distinct professional responsibilities, workplace demands, available resources, and coping capacities. Therefore, the present study examines job‐related stressors across career stages and their relationship with quality‐of‐life enjoyment and satisfaction among nurses in a multicenter setting.

### 1.1 Aim of the Study

The study was conducted to assess job‐related stressors among nurses during different career stages and their relation to quality of life‐enjoyment and satisfaction.

### 1.2 Research Questions

1. What are the levels of job‐related stressors and quality‐of‐life enjoyment and satisfaction among nurses across different career stages?
2. Is there a significant relationship between job‐related stressors and quality‐of‐life enjoyment and satisfaction among nurses across different career stages?

## 2 Methods

### 2.1 Study Design

A multicenter, descriptive‐correlational, cross‐sectional research design was employed in this study in accordance with the *Strengthening the Reporting of Observational Studies in Epidemiology (STROBE)* guidelines. Data collection was conducted across nine public hospitals in Sohag Governorate. The nine hospitals were geographically distributed across different sectors of the governorate, with each hospital located in a distinct geographical area, thereby providing coverage of the governorate's major population centers. These hospitals were selected because they provide a wide range of specialized healthcare services to the local population which reflected in job stressors. Also, it employs a relatively large number of bachelor's‐prepared nurses. This design was selected because the study objectives were primarily descriptive and associational rather than causal. Furthermore, randomization and longitudinal follow‐up were deemed impractical and ethically challenging within the context of multiple hospital settings during the study period.

### 2.2 Sampling and Recruitment

A convenience sample of 277 nurses was recruited from the selected hospitals. The required sample size was estimated based on including an alpha level of 0.05, a medium effect size (*f* ^2^ = 0.15), and a statistical power of 0.80. According to Cohen's guidelines for statistical power analysis, these parameters indicated that a minimum of 184 participants was required to detect meaningful associations (Cohen 1992). The final sample size exceeded this threshold, thereby ensuring adequate statistical power for the planned analyses.

All nurses who fulfilled the inclusion criteria (registered nurses, working in the selected settings, with at least 1 year of clinical experience, and willing to participate) were included in the study. As a result, the sample consisted of 277 nurses overall. The study's participants hold a variety of jobs and have varying years of experience. This strategy sought to obtain the broadest feasible representation of available nursing staff within each hospital, while acknowledging the inherent limitations of convenience sampling, including potential selection bias and limited generalizability.

### 2.3 Study Instruments

#### 2.3.1 General Information Questionnaire

Based on relevant literature (Super 1988; Elliason 2022), and the actual conditions of the institution as well as expert opinions, the questionnaire was self‐designed by the researchers and covered 5 items: age, gender, marital status, role, and career length, which were used to determine participants' career stage.

#### 2.3.2 Work Stress Assessment Questionnaire (WSAQ)

It adopted from Mahmood et al. (2010), was used to assess job‐related stressors. The WSAQ consists of 23 statements covering six domains: Reward, Relation, Demand, Role, Control, Support. Each item is rated on a 5‐point Likert scale ranging from 1 (strongly disagree) to 5 (strongly agree), with higher scores indicating greater perceived job‐related stress. The overall score is calculated as the arithmetic mean of the 23 items, with scores ranging from 1 to 5; a mean score of ≥ 2.5 was considered indicative of job‐related stress. The questionnaire was used in its adopted form without modification. In the present study, the WSAQ demonstrated good internal consistency, with a Cronbach's *α* of 0.89 for the total scale. Because the study examined the individual stressor domains, the WSAQ domains demonstrated satisfactory internal consistency, with Cronbach's *α* coefficients of 0.84 for demands, 0.86 for control, 0.77 for support, 0.83 for role, 0.74 for relationships, and 0.86 for rewards, indicating satisfactory internal consistency across all domains.

#### 2.3.3 Quality‐Of‐Life Enjoyment and Satisfaction Questionnaire‐Short Form (Q‐LES‐Q‐SF)

The Quality of Life Enjoyment and Satisfaction Questionnaire–Short Form (Q‐LES‐Q‐SF), adopted from Bagheri et al. (2022), was utilized to assess participants' satisfaction with general life domains. The instrument comprises 14 items rated on a 5‐point Likert scale (1 = extremely poor to 5 = very good), yielding a maximum score of 70. Domains include physical health, emotions, work, domestic responsibilities, academic activities, leisure, and social relationships, evaluated over the preceding week. Thus, the 14‐item summary score ranges from 14 to 70, with higher scores indicating greater quality‐of‐life enjoyment and satisfaction. Scores are expressed as a percentage of the maximum possible score using the formula:

$$>\text{Percentage}=\frac{\mathrm{Raw}\;\text{Score}-14}{56}\times 100>$$

Scores of 70% or higher fall within the range reported for community samples, whereas scores below 70% indicate lower quality‐of‐life enjoyment and satisfaction relative to this reference range; this should not be interpreted as a stand‐alone clinical diagnosis or universal clinical cut‐off. The Q‐LES‐Q‐SF has demonstrated strong psychometric properties, with internal consistency coefficients 0.86 (Endicott et al. 2011; Stevanovic 2011). Cronbach's alpha was calculated for the Q‐LES‐Q‐SF in the current sample and was *α* = 0.83, indicating acceptable internal consistency.

Instruments validity was established through expert review, whereby a panel evaluated the tools for clarity, relevance, applicability, and ease of implementation. The original English versions of the study instruments were used and administered without translation or modification. To further assess clarity, feasibility, and the time required for data collection, a pilot study was conducted with 28 nurses (representing 10% of the intended sample). Adjustments were made following analysis of the pilot findings, and participants in the pilot study were subsequently excluded from the final sample.

### 2.4 Data Collection Procedure

Following formal authorization from the participating hospitals, data were collected electronically using a structured, self‐administered questionnaire through Google Forms over a 2‐month period from 1 August to 30 September 2025. All nurses who met the predefined eligibility criteria were invited to participate. All eligible nurses were invited, 327 agreed to participate, and 290 completed and submitted the questionnaire, while 13 responses were excluded because they were incomplete data, resulting in a final sample of 277 nurses and a response rate of 88.7%. The survey link was distributed through governorate nursing directors and hospital nursing administration. The questionnaire header provided information about the study purpose, procedures, eligibility criteria, voluntary nature of participation, and confidentiality. Participation was voluntary, and informed consent was implied by submission of the completed questionnaire. Participants were informed that their decision to participate or not participate would have no effect on their employment or relationship with the hospital. To minimize the possibility of duplicate submissions, Google Forms was configured to limit responses to one submission per participant, and submitted responses were screened for potential duplicates based on submission timestamps. No personally identifying information was collected that could be used to link responses to individual participants.

Following data collection, responses were exported to a secure database, checked for completeness and consistency, coded, and prepared for statistical analysis. Data were screened for missing values, and the extent and pattern of missing data were examined before analysis. Missing data were assessed for their extent and pattern, and analyses were conducted using the available data according to the requirements of each statistical procedure.

### 2.5 Ethical Considerations

Ethical approval for this study was obtained from the Research Ethics Committee of the Faculty of Nursing at Sohag University (IRB: 225, March 18th, 2025). Besides that, the study complied with the principles of the Declaration of Helsinki, including respect for participants' autonomy, voluntary participation, the provision of informed consent, assurance of confidentiality and anonymity, the right to withdraw at any time without penalty, and the minimization of potential risks or harm.

Informed consent was obtained electronically from all participants before their participation after they were fully informed about the study objectives, procedures, potential risks and benefits, confidentiality, and their right to withdraw at any time without any consequences. Participants were informed that their participation was voluntary and that their responses would remain confidential and anonymous. Any personal data that reveals their personality has not been collected in the data collection.

### 2.6 Statistical Analysis

Upon completion of data collection, the data were scored, tabulated, and analysed through data entry and analysis by computer using the ‘Statistical Package for Social Science’ (SPSS) version 26. Homogeneity of variance was evaluated using the appropriate tests and graphical inspection where applicable. Independence of observations was ensured through the study design, as each participant provided one independent set of responses. Potential influential outliers were examined using standardized scores and relevant diagnostic measures.

Data were presented using descriptive statistics in the form of percentages, frequency, mean, and standard deviation. Inferential statistical tests of significance, such as Pearson correlation, Chi‐square tests and independent‐samples *t*‐tests and one‐way ANOVA/Welch's ANOVA were used to identify group differences and the relations among the study variables. Effect sizes and 95% confidence intervals were reported where applicable to assess the magnitude and precision of the observed differences.

## 3 Results

As presented in Table 1, the participants were predominantly female (58.5%), with 70.8% aged 25–35 years (mean age = 27.32 ± 6.05 years). Regarding occupational characteristics, 33.9% were engaged in direct nursing care, followed by 33.6% working as head nurse. Beside that, 53.8% had less than 5 years of professional experience, corresponding to the exploration career stage, followed by 34.7% at their establishment stage with 5–10 years of professional experience. More than half of the participants were single (57.4%).

**TABLE 1:** Distribution of personal characteristics of the study participants (*N* = 277).

Characteristic	Category	No.	%
Gender	Male	115	41.5
	Female	162	58.5
Age (years)	< 25	54	19.5
	25–35	196	70.8
	35–45	18	6.5
	> 45	9	3.2
	Mean ± SD	—	27.32 ± 6.05
Role	Direct care nurse	94	33.9
	Head nurse	93	33.6
	Nursing supervisor	60	21.7
	Assistant nurse director	15	5.4
	Nurse director	15	5.4
Career‐stage (years)	Exploration (1–< 5)	149	53.8
	Establishment (5–< 10)	96	34.7
	Advancement (10–< 20)	21	7.6
	Withdrawal (≥ 20)	11	4.0
Marital status	Single	159	57.4
	Married without children	25	9.0
	Married with children	93	33.6

Abbreviation: SD, standard deviation.

The descriptive analysis of job‐related stressors at Table 2 indicated that the Reward domain was perceived as the highest source of stress, with a mean score of 3.94 ± 0.91 followed by the Relation domain at 3.56 ± 0.78 and the Demand domain at 3.60 ± 0.73. Notably, Role (3.64 ± 0.61), Control (3.33 ± 0.80), and Support (3.01 ± 0.84) represented the lowest mean scores, suggesting that these domains were perceived as comparatively less intense stressors relative to Reward, Relation, and Demand. Overall, total job‐related stress was high, with a mean score of 80.91 ± 13.98 corresponding to an average mean score of 4.13 ± 0.70. This reflects a generally elevated level of job‐related stressors among the nursing staff.

**TABLE 2:** Descriptive analysis of job‐related stressors among study participants (*N* = 277).

Domain	No. of items	Mean score (± SD)	Average score (± SD)	Rank
Reward	3	11.82 ± 2.74	3.94 ± 0.91	1
Relation	3	10.69 ± 2.33	3.56 ± 0.78	2
Demand	4	14.39 ± 2.92	3.60 ± 0.73	3
Role	6	21.80 ± 3.64	3.64 ± 0.61	4
Control	4	13.32 ± 6.36	3.33 ± 0.80	5
Support	3	9.04 ± 2.51	3.01 ± 0.84	6
Total	23	80.91 ± 13.98	4.13 ± 0.70	—

The findings at Table 3 related to quality‐of‐life enjoyment and satisfaction revealed notable variations across different domains. Economic status (3.45 ± 1.31) and physical health (3.43 ± 1.06) had the highest mean scores, indicating relatively better satisfaction in these areas. In contrast, leisure time activities (2.29 ± 1.27), overall sense of well‐being (2.51 ± 1.43), and social relationships (2.53 ± 1.45) had the lowest scores, highlighting areas of concern. The total mean score was 2.74 ± 0.33, and 73.65% of participants scored below 70, indicating that most reported unsatisfactory levels of quality‐of‐life enjoyment and satisfaction. These results emphasize the need for targeted interventions to improve psychosocial aspects such as mood, relationships, and leisure activities.

**TABLE 3:** Descriptive quality‐of‐life enjoyment and satisfaction among study participants (*N* = 277).

Domain	Average score (± SD)	Rank
Physical health	3.43 ± 1.06	2
Mood	2.58 ± 1.26	10
Work	2.78 ± 1.41	4
Household activities	2.70 ± 1.48	5
Social relationships	2.53 ± 1.45	12
Family relationships	2.66 ± 1.46	8
Leisure time activities	2.29 ± 1.27	14
Daily functioning	2.64 ± 1.48	9
Sexual life	2.68 ± 1.48	7
Economic status	3.45 ± 1.31	1
Living conditions	2.69 ± 1.47	6
Mobility	2.57 ± 1.46	11
Vision	3.14 ± 1.31	3
Overall well‐being	2.51 ± 1.43	13
Total score	2.74 ± 0.33	—
Score category	No. of responses (%)	
Satisfactory (≥ 70)	73 (26.35%)	
Unsatisfactory (< 70)	204 (73.65%)	

Table 4 demonstrates gender, females reported higher job‐related stress than males, whereas males reported higher quality‐of‐life enjoyment and satisfaction; however, these differences were (*p* = 0.16 and *p* = 0.14, respectively). Significant differences in job‐related stress were observed across age groups, as indicated by Welch's one‐way ANOVA (*F* = 8.53, *p* = 0.04). Similarly, quality‐of‐life enjoyment and satisfaction differed significantly across age groups (Welch's *F* = 11.88, *p* < 0.001). Significant differences in job‐related stress were also observed across nursing roles (Welch's *F* = 11.65, *p* < 0.001), while quality‐of‐life enjoyment and satisfaction differed significantly by role (Welch's *F* = 14.66, *p* < 0.001). In contrast, marital status was not significantly associated with job‐related stress (Welch's *F* = 0.36, *p* = 0.24) or quality‐of‐life enjoyment and satisfaction (Welch's *F* = 0.44, *p* = 0.27).

**TABLE 4:** Relation between personal characteristics, job‐related stress, and Quality of Life Enjoyment and Satisfaction among study participants (*N* = 277).

Personal characteristic	Job‐related stress, Mean ± SD	Test statistic (df)	*p*	Q‐LES‐Q‐SF, Mean ± SD	Test statistic (df)	*p*
Gender						
Male (*n* = 115)	80.58 ± 11.49	*t* = −0.675 (230.52)	0.501	2.84 ± 0.24	*t* = 1.168 (274.83)	0.244
Female (*n* = 162)	81.49 ± 10.43			2.80 ± 0.33		
Age (years)						
< 25 (*n* = 54)	79.68 ± 13.68	Welch's *F* = 6.211 (3, 27.55)	0.002	2.44 ± 0.46	Welch's *F* = 7.074 (3, 49.79)	< 0.001
25–35 (*n* = 196)	73.42 ± 12.65			2.56 ± 0.78		
35–45 (*n* = 18)	68.32 ± 13.05			2.68 ± 0.23		
> 45 (*n* = 9)	63.45 ± 12.46			2.77 ± 0.13		
Role						
Direct care nurse (*n* = 94)	76.43 ± 12.78	Welch's *F* = 8.013 (4, 58.94)	< 0.001	2.32 ± 0.45	Welch's *F* = 13.379 (4, 72.40)	< 0.001
Head nurse (*n* = 93)	74.67 ± 11.05			2.34 ± 0.67		
Nursing supervisor (*n* = 60)	71.66 ± 9.66			2.46 ± 0.87		
Assistant nurse director (*n* = 15)	69.23 ± 10.36			2.68 ± 0.13		
Nurse director (*n* = 15)	65.56 ± 6.21			2.80 ± 0.40		
Career stage						
Exploration (1–< 5 years; *n* = 149)	80.04 ± 13.63	Welch's *F* = 14.087 (3, 37.45)	< 0.001	2.49 ± 0.36	Welch's *F* = 8.790 (3, 33.29)	< 0.001
Establishment (5–< 10 years; *n* = 96)	73.48 ± 10.34			2.68 ± 0.23		
Advancement (10–< 20 years; *n* = 21)	69.35 ± 11.56			2.70 ± 0.45		
Withdrawal (≥ 20 years; *n* = 11)	64.67 ± 8.48			2.89 ± 0.87		
Marital status						
Single (*n* = 159)	77.91 ± 14.45	Welch's *F* = 4.231 (2, 67.11)	0.019	2.69 ± 0.33	Welch's *F* = 3.935 (2, 165.14)	0.021
Married without children (*n* = 25)	71.67 ± 13.35			2.66 ± 0.05		
Married with children (*n* = 93)	73.67 ± 12.23			2.83 ± 0.61		

*Note:* *F*, Welch's ANOVA test; *t*, independent *t*‐test.

Job‐related stress progressively declined as participants advanced through their careers, from 80.04 ± 13.63 during the exploration stage (1–< 5 years) to 73.48 ± 10.34 during establishment (5–< 10 years), 69.35 ± 11.56 during advancement (10–< 20 years), and 64.67 ± 8.48 during withdrawal (≥ 20 years). Conversely, quality‐of‐life enjoyment and satisfaction increased steadily across the same career stages, from 2.49 ± 0.36 during exploration to 2.68 ± 0.23 during establishment, 2.70 ± 0.45 during advancement, and 2.89 ± 0.87 during withdrawal. Across career stages, job‐related stress differed significantly (Welch's *F* = 9.51, *p* = 0.04), while quality‐of‐life enjoyment and satisfaction also differed significantly (Welch's *F* = 13.69, *p* < 0.001). Thus, the findings indicate that career progression was associated with a gradual reduction in job‐related stress and a corresponding improvement in quality‐of‐life enjoyment and satisfaction, suggesting that accumulated professional experience and career maturity may contribute to greater occupational adaptation and overall well‐being.

Finally, the results revealed a strong and statistically significant negative correlation between total job‐related stress and overall quality‐of‐life enjoyment and satisfaction (*r* = −0.83, *p* < 0.001). This indicates that as job‐related stress increases, nurses' Quality of Life Enjoyment and Satisfaction decrease markedly. The strength of this relationship underscores the substantial impact of occupational stressors on well‐being and highlights the importance of effective stress‐management strategies to enhance nurses' Quality of Life Enjoyment and Satisfaction. See Table 5.

**TABLE 5:** Correlation between job‐related stress and Quality of Life Enjoyment and Satisfaction (*N* = 277).

Variable	*r*	*p*
	Quality of Life Enjoyment and Satisfaction	
Job‐related stress v	−0.83	< 0.001

## 4 Discussion

Stress is a part of nurses' everyday life and affects all aspects of our activities. The nature of the nursing profession encounters many types of stressors throughout the career path (Abbaszadeh et al. 2024). The study was designed to assess job‐related stressors and their relation to quality‐of‐life enjoyment and satisfaction among nurses during different career stages. The findings highlight the multifaceted nature of job‐related stressors among participant nurses and its significant negative effect on their quality‐of‐life enjoyment and satisfaction.

Regarding personal characteristics of the participants, it was shown that more than half of the study participants were females and about two‐thirds of their ages varied from 25 to 35 years. More than half of the study participants were at their exploration stage and about a third of them were at their establishment career stage. Nearly one‐third of them were working as head nurses, and another third of them were providing direct nursing care. More than half of the participants were single, and the rest of them were married. These demographic characteristics are consistent with nursing workforce trends in many healthcare systems in Egypt, where younger nurses often dominate frontline care. Such characteristics may predispose nurses to higher stress levels, particularly when combined with limited professional experience and social support.

Regarding descriptive analysis of the studied variables, Table 2 reveals that Reward was perceived as the most significant stressor, followed by Relation and Demand, while Role and Control, Support were perceived as comparatively less intense stressors. These findings can be interpreted through the Job Demands–Resources (JD–R) model, which proposes that job demands contribute to strain, while adequate job resources can help employees manage these demands and protect their well‐being. This finding aligns with previous studies such as Alahiane et al. (2023) in a study on 223 nurses in Morocco, who reported that reward was associated with nurses' psychological health, job satisfaction, and quality of life and nurses' well‐being. Shi et al. (2024), in a study of 696 Chinese nurses, found that effort–reward imbalance was an important factor associated with nurses' perceived stress. Similarly, Zhang et al. (2024) reported that effort–reward imbalance was associated with poorer well‐being among psychiatric nurses, while Braun et al. (2024) demonstrated a significant association between effort–reward imbalance and burnout risk among emergency nurses and emphasized the potential importance of salary and recognition as forms of reward.

In contrast, a study among nurses in Iranian public hospitals (Babamiri et al. 2022) found that the Demand–Control–Support model had greater predictive power than the Effort–Reward Imbalance model, suggesting that demand, control, and social support may be more influential work‐related factors than reward in some nursing settings. Also, An Indian study about occupational stress, mental health, and coping among information technology professionals revealed that job demand and superior control had the main effect on work stress level, which was incongruent with this study's result (Elliason 2022). This discrepancy may be explained by differences in the clinical context and characteristics of the nursing population, variations in organizational settings, nursing roles, workload, managerial practices, and perceived autonomy and support across healthcare systems.

The current study found a relatively high overall level of job‐related stress among nurses, as reflected by the total mean score of 80.91 ± 13.98. This finding indicates that nurses were exposed to substantial work‐related stressors, which reflect the complex nature of nursing work, including high workload, interpersonal demands, responsibility for patient care, and perceived inadequacies in recognition or compensation. This finding highlights the need for organizational interventions aimed at reducing occupational stress and improving nurses' working conditions and well‐being. The present finding is consistent with Okuhara et al. (2021), who reported high levels of occupational stress among healthcare workers and highlighted the substantial impact of work‐related stressors on employee well‐being and work‐related outcomes. In contrast, Manshor et al. (2019) reported moderate to low levels of occupational stress among managers in Malaysia. This discrepancy may be attributable to differences in professional roles, work responsibilities, organizational environments, staffing conditions, healthcare systems, study populations, and stress measurement approaches. Therefore, the interpretation of the present stress score should be considered within the context of the specific instrument, study population, and healthcare setting. Overall, the findings emphasize the importance of context‐specific organizational interventions to address job‐related stress among nurses.

Table 3 highlights substantial variation in quality‐of‐life enjoyment and satisfaction. Economic status and Physical health ranked highest, while leisure activities, overall well‐being, and social relationships scored lowest. The majority of participants reported unsatisfactory levels of quality‐of‐life enjoyment and satisfaction, which may be explained by the JD–R model, which suggests that persistent job demands and insufficient resources can deplete nurses' well‐being. Consistent with this perspective, Shdaifat et al. (2023) found that work‐related stress was directly associated with burnout and secondary traumatic stress among nurses, while job satisfaction and compassion satisfaction were related to professional quality of life.

Limited leisure time and strained social relationships may exacerbate stress and reduce nurses' resilience to save their optimal level of Quality of Life Enjoyment and Satisfaction. Similar findings were reported by Lalkhaida et al. (2022), who found that economic stability and physical functioning were relatively preserved, whereas psychosocial domains, including emotional well‐being and interpersonal relationships, were more adversely affected by occupational stress. A study conducted among 1179 district‐hospital nurses in Vietnam using the Q‐LES‐Q‐SF found that nurses experiencing musculoskeletal symptoms and psychological distress had poorer quality of life (Nguyen et al. 2020). Also, Dutta and Khanikor (2022), who reported that more than half of the participants had a low Quality of Life Enjoyment and Satisfaction. This may support the present finding, as persistent occupational stress and associated physical and psychological burdens can negatively affect enjoyment and satisfaction across different areas of daily life.

Career stage was significantly associated with both job‐related stress and quality‐of‐life enjoyment and satisfaction. Nurses in the exploration stage reported the highest job‐related stress and the lowest Q‐LES‐Q‐SF mean score, whereas those in the withdrawal stage reported the lowest stress and the highest Q‐LES‐Q‐SF mean score. This pattern indicates that nurses with longer tenure tended to report lower stress and better quality of life. A similar association was reported by Mukhlis et al. (2024), who found significantly better quality of life among employees with more than 5 years of service compared with those with 1–3 years of service. These findings provide supporting evidence for the relationship between longer tenure and better quality of life. The observed differences may be related to accumulated occupational experience and greater familiarity with workplace demands over the course of a nursing career.

Also, this result may be explained from a JD–R perspective; the higher stress reported by nurses in the exploration stage may reflect relatively lower autonomy and control, developing professional confidence, and fewer established organizational resources, which may make managing job demands more challenging. In contrast, more experienced nurses may benefit from greater professional autonomy and familiarity with their roles, although accumulated physical demands and concerns regarding career progression may continue to affect their well‐being. This interpretation is supported by recent evidence showing that early‐ and mid‐career nurses experience poorer well‐being and greater fatigue than nurses at other career stages, while job demands and insufficient resources are important determinants of nurses' mental health (Cuccia et al. 2026). According to Balakrishnan and JohnCecily (2026), individual characteristics, including nurses' career stage and personal circumstances, play a significant role in how they manage the competing demands of work and family life.

The correlational analyses provide deeper insights into the interplay between personal characteristics, career stages, job‐related stress, and Quality of Life Enjoyment and Satisfaction among study participants. Age, role, and career stage were negatively correlated with stress but positively associated with quality‐of‐life satisfaction, suggesting that professional maturity and role stability buffer against occupational strain. Gender and marital status, however, showed no significant associations, indicating that demographic factors may be less influential than career‐related variables.

It was observed that age, role, and career length were negatively correlated with stress but positively correlated with Quality of Life Enjoyment and Satisfaction. This finding is consistent with Kerksieck et al. (2024), who reported that greater experience and more effective work‐nonwork balance strategies were associated with improved well‐being and coping capacity. Gender and marital status were not significant in the present study, which supports evidence suggesting that career‐related factors may have a stronger influence on occupational stress and well‐being than demographic characteristics. These findings highlight the importance of considering career progression and professional experience when developing interventions to improve nurses' Quality of Life Enjoyment and Satisfaction and reduce work‐related stress.

The level of job‐related stress varied according to participants' personal characteristics. A moderate, non‐significant positive correlation was observed between gender and job‐related stress. Males and females may respond differently to workplace stressors. In nursing, which has traditionally been viewed as a female‐dominated profession, male nurses may experience additional stress related to workplace integration and role expectations. In contrast, Lai et al. (2020) reported that the work‐home interface was particularly challenging for female healthcare workers, who often bear greater family and childcare responsibilities, thereby increasing their psychological stress burden.

The study revealed a moderate, non‐significant positive correlation between marital status and the level of job‐related stress. This finding may be explained by the additional responsibilities faced by married nurses, who must balance work demands with family obligations and financial commitments. Long working hours may reduce time spent with partners, children, family, and friends, potentially increasing stress and work–family conflict. Similar findings were reported by Gmayinaam et al. (2024), who identified workplace demands and challenging working conditions as important contributors to occupational stress among nurses. These factors may place additional burdens on married nurses as they attempt to balance professional and family roles.

Besides that, the present study showed a strong negative correlation between age, role, career stage, and job‐related stress, indicating that nurses in the establishment stage experienced the highest levels of work stress. According to Super's career development theory (Super 1988), the establishment stage represents a critical phase of career development during which individuals strive to stabilize their professional roles and pursue advancement opportunities. As responsibilities increase during this stage, nurses may experience greater occupational stress associated with performance expectations and career progression. Similar findings were reported by Stašević et al. (2025), who found that professional demands and workplace stressors were associated with elevated levels of stress among nurses.

Table 5 revealed a strong negative correlation between job‐related stress and overall quality‐of‐life enjoyment and satisfaction, underscoring the profound impact of occupational stress on nurses' well‐being. This relationship may be explained by the cumulative effects of workload, interpersonal challenges, and limited resources, which can adversely affect nurses' well‐being, caring behaviours, and professional functioning. These findings are consistent with those of Babapour et al. (2022) found that higher job stress was significantly associated with poorer Quality of Life Enjoyment and Satisfaction and reduced caring behaviours. Similar results were reported by Dartey et al. (2023), who concluded that occupational stress negatively affects nurses' physical and psychosocial well‐being, diminishing resilience and work performance. From the PERMA perspective, sustained occupational stress may negatively affect Positive emotions, Engagement, Relationships, Meaning, and Accomplishment, thereby reducing nurses' overall enjoyment and satisfaction with life. Recent nursing research similarly highlights organizational support, professional development, recognition, and supportive relationships as important contributors to nurses' flourishing and well‐being (Bond et al. 2025).

Deside that, Balakrishnan and JohnCecily (2026), reported in their study that nurses at different career stages may experience distinct work–life balance challenges and require tailored organizational support to effectively cope with these demands. Beside that, Kupcewicz et al. (2025), who reported that nurses in earlier career stages experience greater stress due to role ambiguity, high workload, and limited coping resources, while more experienced nurses tend to develop stronger coping abilities, resilience, and role clarity. The consistency of these findings strengthens the evidence that reducing occupational stress is essential for improving nurses' Quality of Life Enjoyment and Satisfaction and professional outcomes.

## 5 Implications for Nursing & Health Policy

The study findings support creating policies that institutionalize stress management, tailor interventions by career stage, enhance psychosocial well‐being, and reshape the public image of nursing, such as the Stress Reduction Policy which mandates structured reward systems (fair pay, recognition programs, promotion pathways), requires clear role descriptions and supportive supervision to reduce role ambiguity, and implements flexible scheduling and workload redistribution to protect nurses' work‐life balance. Also, Career Stage–Specific Policy, which involves developing targeted support programs for early‐ and mid‐career nurses (mentorship, resilience training), provides advancement opportunities and leadership development for mid‐career nurses to prevent burnout, and introduces wellness and adaptation programs for late‐career nurses to sustain engagement. Quality of Life Enjoyment and Satisfaction Policy that can emphasizes the systematic evaluation of nurses' well‐being across multiple life domains, including physical health, emotional stability, social relationships, occupational functioning, and leisure activities. Finally, the Monitoring & Evaluation Policy establishes regular assessments of job‐related stress and Quality of Life Enjoyment and Satisfaction using validated tools and requires hospital managers to report annually on nurse well‐being indicators.

## 6 Conclusion

The present study concluded that nurses experienced high levels of job‐related stress, with Reward identified as the most significant stressor, with overall elevated stress scores. Quality‐of‐life enjoyment and satisfaction domains showed marked variation, with physical health and economic status ranking highest; more than two‐thirds of participants reported unsatisfactory quality‐of‐life enjoyment and satisfaction. Stress and dissatisfaction peaked during the establishment and exploration stages. The majority of participants had a high level of job‐related stressors and a low level of quality‐of‐life enjoyment and satisfaction. There was a strong negative correlation was found between total job‐related stress and overall quality‐of‐life satisfaction, highlighting the association of job‐related stress on nurses' quality‐of‐life enjoyment and satisfaction and the need for targeted interventions, particularly for early‐ and mid‐career nurses. This knowledge is important for health managers to control the sources of work‐related stress, then employees are more committed and provide quality care, which increases the reputation and prestige of an organization.

## 7 Limitations

First, the cross‐sectional design precludes establishing causal relationships between studied variables. Second, the use of convenience sampling may have introduced selection bias, while the study recruited nurses from multiple public hospitals and applied predefined eligibility criteria consistently across all participating sites to eliminate selection bias or enhance the generalizability. Third, the career‐stage groups were unequal in size, particularly the small number of nurses in the withdrawal stage (*n* = 11), which may have limited the statistical power to detect differences between groups. Fourth, the use of instruments that were not specifically adapted to all characteristics of the study context may have introduced measurement limitations, despite their established reliability and validity. Finally, potential non‐response bias cannot be excluded because nurses who did not participate or submitted incomplete responses may have differed systematically from participants. Therefore, the findings should be interpreted with caution and confirmed through larger, more representative, and preferably longitudinal studies with more balanced career‐stage groups.

## 8 Recommendations

The findings suggested further research that focuses on the personality traits of an individual to cope with job stress. Conduct long‐term follow‐up studies to track how job‐related stress and Quality of Life Enjoyment and Satisfaction evolve across different career stages, rather than relying on cross‐sectional data. Compare stress and satisfaction levels among nurses working in different healthcare settings (public vs. private hospitals, urban vs. rural facilities) to identify contextual factors influencing outcomes. Investigate in more depth how gender roles and family responsibilities interact with occupational stress, particularly in societies where nursing is perceived as a female‐dominated profession. Explore how cultural perceptions of nursing and societal respect impact nurses' satisfaction and career retention across different regions or countries.

## Author Contributions

**Hind Ismail Ali:** conceptualization, validation, formal analysis, project administration, funding acquisition. **Safaa Moustafa Mohamed Abdeen:** formal analysis, writing – review and editing, writing – original draft, methodology. **Hanaa Sayed Ewise:** software, visualization, writing – review and editing, writing – original draft.

## Funding

This research was funded by Prince Sattam bin Abdulaziz University through Project Number (PSAU/2025/01/36694). The funding body had no role in the study design, data collection, analysis, interpretation of data, manuscript preparation, or the decision to publish the results.

## Ethics Statement

Ethical approval for this study was obtained from the Research Ethics Committee of the Faculty of Nursing at Sohag University (IRB: 225, March 18th, 2025). Besides that, the study complied with the principles of the Declaration of Helsinki.

## Consent

The authors have nothing to report.

## Conflicts of Interest

The authors declare no conflicts of interest.

## Data Availability

The data that support the findings of the study are available from the corresponding author upon reasonable request.
